# Meningitis diagnosis, treatment, and outcomes in rural, northern Uganda: 2015–2024

**DOI:** 10.1371/journal.pgph.0005800

**Published:** 2026-01-12

**Authors:** Abigail Link, Mark Okwir, Betty Nabongo, Fred Okello, Jimmy Alal, David Meya, Paul R. Bohjanen

**Affiliations:** 1 Division of Infectious Diseases, Department of Medicine, School of Medicine and Dentistry, University of Rochester, Rochester, New York, United States of America; 2 MARA-Rural Health Consortium, Lira, Uganda; 3 Department of Public Health Sciences, School of Medicine and Dentistry, University of Rochester, Rochester, New York, United States of America; 4 Department of Internal Medicine, Lira University, Lira, Uganda; 5 Lira Regional Referral Hospital, Lira, Uganda; 6 Infectious Diseases Institute, Makerere University, Kampala, Uganda; 7 Department of Medicine, College of Health Sciences, Makerere University, Kampala, Uganda; 8 Department of Medicine, Infectious Diseases and International Medicine, University of Minnesota, Minneapolis, Minnesota, United States of America; UMass Chan Medical School - Baystate Medical Center, UNITED STATES OF AMERICA

## Abstract

Meningitis affects over 2.5 million people worldwide, primarily within resource-limited regions of the meningitis belt of Africa. In 2017, we implemented a cryptococcal meningitis (CM) diagnosis and treatment program (CM-DTP) designed to improve CM outcomes. In 2021, a meningitis diagnosis and treatment program (MEN-DTP) began to include all etiologies of meningitis to assess the impact of expanded diagnosis and treatment of meningitis. We conducted a retrospective study utilizing clinical records from 1443 adult patients admitted to Lira Regional Referral Hospital (LRRH) over three time periods between 2015–2024. Group 1 included 321 patients in a historical control group; Group 2 consisted of 890 patients during the CM-DTP and Group 3 included 232 patients during the MEN-DTP. Group 1 received routine meningitis care, Group 2 received testing and treatment focused on CM, and Group 3 received expanded diagnostic testing, including gram stain, culture, PCR- and antigen-based testing. Meningitis diagnosis and in-hospital mortality outcomes were assessed to evaluate our programs. A confirmed meningitis etiology was found in 13.7% in Group 1, 20.7% in Group 2, and 42.2% in Group 3. In Group 3, confirmed etiologies were identified based on culture (n = 30), Pastorex LA (n = 23), BioFire PCR (n = 56), GeneXpert Ultra (n = 14), and serum or CSF CrAg LFA (n = 53). Overall, more confirmed etiologies of meningitis were identified among people living with HIV (PLWH) (n = 319) compared to those without HIV (n = 22) in Group 3. Antibiotic use increased with pre-admission antibiotic use doubling from Group 1 to Group 3 (10.6% to 27.2%). Compared to Group 1, mortality improved in Groups 2 and 3. Overall, PLWH had increased mortality compared to those without HIV (32.4% vs. 13.3%). Introduction of molecular diagnostics increased meningitis diagnoses and improved outcomes, particularly in those with CM. MEN-DTP increased confirmed diagnoses, yet half remained undiagnosed, supporting investigation of deep sequencing technologies.

## Introduction

Meningitis is an often a deadly inflammation of the meninges, the linings of the brain, which can be caused by various pathogens and occurs in over 2.5 million people worldwide each year [1]. However, due to limited surveillance and monitoring in some countries, this figure may be underestimated. Meningitis is caused by several types of bacteria, viruses, fungi, or parasites [2]. Bacterial meningitis (BM) can be treated with antibiotics, and some forms of BM can be prevented with vaccines. In people living with HIV (PLWH), the fungus *Cryptococcus neoformans* is a major cause of meningitis [3], which is often fatal. The “meningitis belt” region of Africa, which includes Uganda along with 25 other countries spreads from Senegal to Ethiopia, and has the highest rates of meningitis in the world [4,5]. Seasonal epidemics of BM are common, which typically occur from November to June (dry season) [6,7]. Health care resources and infrastructure in many parts of the meningitis belt, especially rural regions, are suboptimal making meningitis diagnosis and care challenging.

To improve meningitis care in rural, northern Uganda, a low-resource region with high prevalence of HIV, our team implemented a CM diagnosis and treatment program (CM-DTP) in 2017 at Lira Regional Referral Hospital (LRRH), a Ugandan government hospital. Our initial focus concentrated on the diagnosis and treatment of CM, a major cause of fatal meningitis, because resources for diagnosis and treatment for CM were lacking in the region. This program led to increased diagnosis of CM, initiation of appropriate treatment and improved meningitis outcomes [8]. In 2021, we expanded diagnostic testing and implemented a broader meningitis diagnosis and treatment program (MEN-DTP) to include additional diagnostic testing for BM, tuberculosis meningitis (TBM), and viral meningitis (VM), utilizing molecular methods based on PCR or antigen detection.

Little is known about the true prevalence, etiologies, and outcomes of meningitis in rural, northern Uganda, which lies within the meningitis belt of Africa. The expanded MEN-DTP was designed to improve meningitis diagnosis and outcomes. Here, we evaluate the impact of expanded diagnostic testing through the MEN-DTP on understanding the etiologies of meningitis, the burden of disease, and patient outcomes.

## Methods

### Setting

This study was conducted at LRRH, a primary government referral center serving over 2.2 million people in northern Uganda, admitting 1,300 patients each month [9], and located in the region of Uganda with the second highest HIV prevalence (7.6%) [10]. Currently, routine vaccines for meningitis in northern Uganda are only available for the pediatric population (Hib and pneumococcal) [11], but are not available for adults outside of epidemic settings.

### Ethics statement

Ethics approval was obtained from Gulu University Research Ethics Committee [GUREC-066-19), Mbarara University of Science and Technology Research Ethics Committee [MUREC 1/7], Uganda National Council of Science and Technology [HS1420ES], University of Minnesota Institutional Review Board [STUDY000012864], and the University of Rochester Institutional Review Board [STUDY00011386]. Secondary data was collected from hospital charts and databases along with clinical record forms between January 10, 2024, to March 30, 2024. Source documents with identifiable participant information was de-identified during data extraction and analysis.

### Patient population and study design

We conducted a retrospective study utilizing hospital records and clinical records forms from 1443 adult patients with meningitis admitted to LRRH from 2015 to 2024. Patients were identified with meningitis based on available diagnostic testing or clinical criteria including headache or irritability in combination with fever, confusion, or Glasgow coma scale (GCS) <14, photophobia, neck pain/stiffness, or seizure. Patients with meningitis were placed into three groups based on the dates of their admission: 321 patients were a historical control group admitted from 2015-2017, prior to initiation of our meningitis programs (Group 1), 890 patients were admitted from 2017-2021 when the CM-DTP was in place (Group 2), and 232 patients were admitted from 2021-2024 during the expanded MEN-DTP (Group 3). All patients with meningitis were included for each time period, thus representing the entire population of patients admitted to LRRH during these periods. Data was collected on clinical record forms at the time of their care from patients who participated in the CM-DTP (Group 2) or MEN-DTP (Group 3) related to diagnosis, symptoms, symptom days, treatments, medical history, physical exams, laboratory results, HIV and antiretroviral therapy (ART) status, complications, diagnosis, treatment, and mortality outcomes. For patients in Group 1, similar data was collected retrospectively from hospital charts. All information was entered into a database, which was validated and double-checked by a second person.

We identified patients to have a confirmed meningitis diagnosis if they had positive results in CSF culture, Pastorex, BioFire, India Ink, GeneXpert, CrAg LFA, or gram stain. Presumed CM (predominantly in Group 1) was defined as a suspected clinical diagnosis of CM with treatment for CM but without a positive confirmatory test. Presumed BM was defined as suspected BM among those who received treatment for BM with a CSF cell count (≥30 cells/mm^3^) and predominantly polymorphonucleocyte pleocytosis, but without a positive confirmatory test. Presumed VM was defined by a CSF cell count (≥30 cells/mm^3^) with predominantly lymphocytic pleocytosis but no positive confirmation test (BioFire). Presumed TBM was designated among meningitis patients with clinical TB or a positive urine LAM, but a negative CSF GeneXpert. The etiology of meningitis was considered unknown in patients without a confirmed or presumed diagnosis.

#### Group 1 (2015–2017).

Group 1 represented a historical group of patients with meningitis admitted to LRRH from 2015 to 2017, prior to initiation of the CM-DTP in 2017. Between 2015–17 meningitis diagnostic testing was limited, and CrAg lateral flow assay (LFA) tests were sporadically available through the government medical supply network. Other CSF testing, including CSF cell count, Ziehl-Neelsen (ZN) stain, India ink, and CrAg, were only performed at private-pay laboratories when patients were able to afford testing. Very few cases had lumbar punctures performed or documented in the hospital chart and most were treated solely on symptomology and clinical judgment (S1 Table; Group 1). Additionally, treatment for CM was often limited as antifungal medications such as amphotericin B deoxycholate and fluconazole were expensive and difficult to procure. However, for patients who were able to afford and receive amphotericin B deoxycholate, monitoring of renal function and electrolyte levels was often inadequate, and electrolyte replacement was rarely provided which led to poorer outcomes. In summary, many patients did not receive adequate treatment or monitoring for CM.

#### Group 2 (2017–2021).

The CM-DTP began in 2017 (Group 2) and provided CrAg LFA strips (IMMY, Norman, Oklahoma, USA) for serum and CSF testing to diagnose CM. As previously described, The CM-DTP provided amphotericin B deoxycholate, fluconazole, routine laboratory monitoring and testing, therapeutic lumbar punctures, daily monitoring while inpatient, and routine outpatient follow-up with fluconazole refills. CSF analysis including cell count was conducted at a private lab when paid for by the patients (S1 Table; Group 2). Empiric treatment for BM consisted of ceftriaxone.

#### Group 3 (2021–2024).

The MEN-DTP included all the supplies and laboratory support from Group 2 with additional tests including CSF cell count, gram stain, culture, Ziehl-Neelsen stain, India ink, TB GeneXpert Ultra (Cepheid, Sunnyvale, CA, USA), Pastorex LA (Bio-Rad, Hercules, CA, USA) to identify bacterial causes (S2 Table), and BioFire meningitis/encephalitis panel PCR (Biomérieux, Marcy-l’Étoile, France) which identifies common bacterial, fungal, and viral pathogens (S2 Table, left). BioFire testing was performed on batched frozen samples sent to the Infectious Diseases Institute in Kampala, Uganda, which prevented the results from impacting care in real time. All other testing was performed at LRRH or private labs in Lira, Uganda. The combination of these diagnostic tests provided a more comprehensive identification of different pathogens than was previously available (S1 Table; Group 3).

### Treatment for meningitis

In all groups, patients typically received ceftriaxone as empiric treatment for BM when they presented with signs and symptoms of meningitis (S1 Table). In Group 1, this was often the only treatment since availability of diagnostic testing was limited. For some patients with suspected CM, empiric antifungal treatment with fluconazole or occasionally amphotericin B deoxycholate was given. In all groups, empiric antiviral therapy was rarely given due to limited supplies and lack of a confirmed diagnosis.

Treatment of CM consisted of deoxycholate amphotericin B, liposomal amphotericin B, flucytosine and fluconazole per Ugandan guidelines, which evolved over time [12]. Empiric or targeted treatment of BM utilized ceftriaxone with or without steroids. TBM was treated with standard anti-TB regimens, including steroids. In a small number of cases, acyclovir was used for empiric treatment of viral meningitis.

### Analysis

The primary outcome of interest was meningitis etiology in each group and the secondary outcome was in-hospital mortality. Descriptive statistics, using means and proportions compared each of the groups to analyze demographic information, diagnosis, diagnostics used, HIV and TB history, and mortality. Evaluating the relationships between the groups, Chi-square test or Fisher’s Exact test was performed for categorical variables and the Student’s t-test was used for continuous variables. Hypothesis testing was two-sided with a significance level of 0.05. All analyses were performed using R version 4.4.3 [13].

## Results

Three Groups were identified (see methods) with the mean age between 37 and 39 years for all groups and a similar distribution of males and females across the groups (Table 1). Fewer patients were living with HIV or had a history of TB in Group 3 compared to Groups 1 and 2. The number of symptoms days before seeking care at LRRH ranged between 13.9-19.4 days. Patients in Group 3 were hospitalized longer (16 days) compared to Groups 1 and 2 (8 and 12 days). More patients were referred to LRRH from outside facilities in Group 3 and the use of antibiotics progressively increased over time. In particular, pre-admission exposure to antibiotics doubled from Group 1 to Group 3 (10.5% to 27.2%), and this pre-exposure to antibiotics would be expected to prevent diagnosis of BM based on CSF culture results [14].

**Table 1:** Demographics and baseline data.

	GROUP 1	GROUP 2	GROUP 3	
	2015-2017 (JAN-FEB 12)	2017-2021 (FEB 13-SEPT 20)	2021-2024 (SEPT 21-JAN 30)	TOTAL
	**n = 321**	**n = 890**	**n = 232**	**n = 1443**
**Sex Female (n, %)**	155 (48.3)	498 (56.0)	98 (42.2)	751 (52.0)
**Age (mean, IQR)**	38.1 (30-45)	39.1 (30-47)	37.0, (30-46.3)	38.1 (30-46)
	**n (%)**	**n (%)**	**n (%)**	**n (%)**
**HIV Positive**	266 (82.9)	748 (84.0)	166 (71.6)	1180 (81.8)
**TB History**	65 (20.2)	149 (16.7)	28 (12.1)	242 (16.8)
**Referred to LRRH**	134 (41.7)	323 (36.3)	114 (49.1)	571 (39.6)
**Referred from**				
Health center II	2 (0.60)	5 (0.60)	1 (0.43)	8 (0.55)
Health center III/clinic	54 (16.8)	150 (16.9)	57 (24.6)	261 (18.1)
Health center IV/hospital	31 (9.7)	94 (10.6)	56 (24.1)	181 (12.5)
HIV clinic	47 (14.6)	66 (7.4)	0	113 (7.8)
**Pre-Admission antibiotic treatment**	34 (10.6)	107 (12.0)	63 (27.2)	204 (14.1)
	**mean, IQR**	**mean, IQR**	**mean, IQR**	**mean, IQR**
**CD4 count**	141.6 (25.8-187.8)	220.8 (33.5-333)	221.8 (39.5-390.0)	194.9 (32.8-297)
**GCS on admission**	13.5 (13-15)	13.2 (12-15)	12.8 (12-15)	13.2 (12.3-15)
**Hospital days**	8 (4-11)	12.4 (5-15.5)	16.3 (7-20)	12.2 (5.3-15.5)
**Symptom days**	13.9 (3-14)	19.4 (5-21)	16.6 (5-21)	16.5 (4.3-18.7)

TB: tuberculosis; GCS: Glascow coma scale.

### Diagnostics

#### Increased utilization of meningitis diagnostic tests over time.

The availability and utilization of meningitis diagnostic tests increased over the 9-year period (S1 Table) In Group 1, few patients were tested for CM and even fewer were tested for other forms of meningitis due to insufficient diagnostic resources at LRRH and the high testing costs at private laboratories. This finding was exemplified in Group 1, as only 35.5% had CSF CrAg testing, whereas after initiation of the CM-DTP and MEN-DTP, 46.4% and 67.7.% of patients had CSF CrAg testing in Groups 2 and 3, respectively. Overall, CSF CrAg tests (IMMY, Norman, OK, USA), identified more patients with CM (n = 234) compared to culture (n = 25) and India Ink (n = 106) including all groups. CSF culture was not available in Group 1 and rarely utilized in Group 2, but for Group 3, CSF culture was performed in 88.9% of patients through improved microbiology laboratory infrastructure at LRRH. CSF cell counts were not available in Group 1, but 46.3% and 66.3% of patients had cell counts performed in Groups 2 and 3, respectively. In Group 3, molecular tests consisting of Pastorex, BioFire and TB GeneXpert PCR-based tests were introduced which enabled detection of several etiologies of meningitis, including BM, VM, and TBM. (S2 Table)

### Comparison of diagnostic tests for meningitis

#### Molecular diagnostics outperformed CSF culture in Group 3.

**Fig 1:**
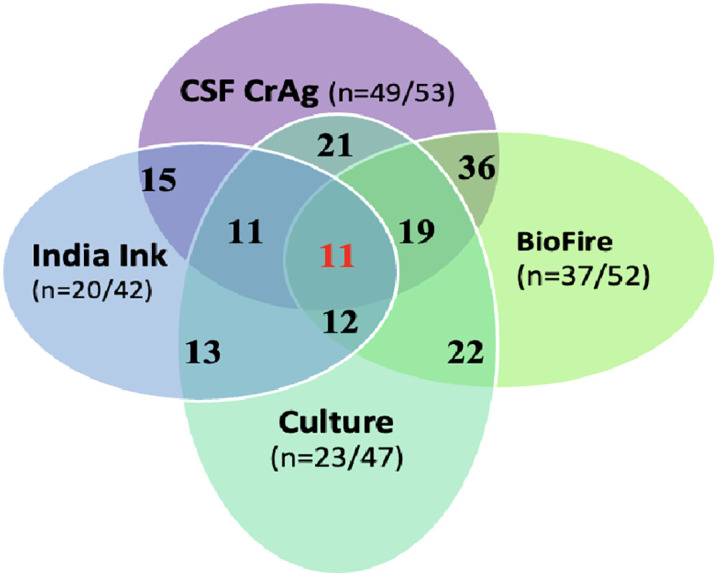
Diagnostic tests in Group 3 that confirmed CM.

The addition of molecular diagnostics in Group 3 improved the identification of meningitis etiologies. Among participants in Group 3 tested for CM, CSF CrAg was superior to BioFire and culture, identifying 49/53 (92.5%) of confirmed cases, whereas BioFire and culture identified 72.5% and 50.0% of confirmed CM cases, respectively (Fig 1). CSF CrAg had high positive result congruency at 97.3% (36/37) with BioFire and 91.3% (21/23) with culture. CSF culture, India ink, BioFire, and CrAg LFA can all detect Cryptococcus; however, molecular diagnostic tests (CrAg, BIoFire) outperformed standard tests such as India Ink and culture for CM identification (Table 2).

**Table 2:** Diagnostics utilized for CM in Group 3.

Diagnostic	India Ink n = 42	Culture n = 47	CrAg LFA CSF n = 53	BioFire n = 52
	Positiven (%)	Positive n (%)	Positive n (%)	Positiven (%)
India Ink	20 (47.6)			
Culture	13 (31.0)	23 (48.9)		
CSF CrAg LFA	15 (35.7)	21 (44.7)	49(92.5)	
BioFire	16 (38.1)	22 (46.8)	36 (67.9)	37 (71.1)

CSF: cerebral spinal fluid; CrAg: cryptococcal antigen.

Comparing positive cryptococcal culture to India ink, we found slightly more CM cases were identified by culture (48.9%) compared to India ink (47.6%), with only 31% congruency between the two tests. Relying on culture (n = 23/53) or India ink (20/53) alone for CM identification, would have missed 50% of the true CM cases. Despite the limitations of culture and India ink, these methods identified some cases which were not identified by CrAg (Fig 1). Our evaluation of the four tests used to identify Cryptococcus highlighted the lack of congruency between the tests, which suggests that more than one test should be considered for comprehensive diagnosis of CM.

BioFire was the only test available in our program to identify viruses. Frozen, batched samples were shipped to Kampala for testing, thus, real-time results for diagnosis and treatment were not possible. Fourteen cases of viral meningitis were identified using BioFire, which would have been previously undiagnosed, but only 20% were empirically treated with antivirals. Also, introduction of TB GeneXpert performed on CSF allowed for the identification of 15 cases of TBM. We found multiple cases where the same diagnosis was made with more than one test although results were often incongruous; thus, utilization of all four testing modalities (culture, Pastorex, BioFire, GeneXpert) was advantageous and provided the highest diagnostic yield.

#### Confirmed meningitis diagnoses improved over time.

**Fig 2:**
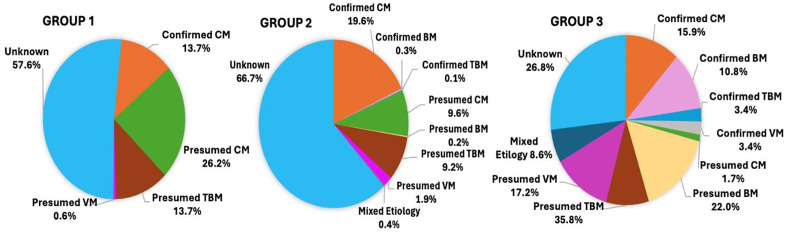
Meningitis etiologies in Groups 1-3.

Group 1 had few confirmed meningitis cases (13.7%), but the number of confirmed diagnoses increased with each subsequent group with the most identified in Group 3 (42.2%) (Table 3 and Fig 2). We identified 272 confirmed cases of CM over the 9-year period with the percentage of confirmed CM among all meningitis cases nearly doubling from 13.7% in Group 1 to 22.4% in Group 3. We found CM was the most identified meningitis etiology during each period. Presumed CM cases steadily decreased from 26.2% in Group 1 to 1.7% in Group 3 as CrAg testing became widely available, and the number of confirmed cryptococcal meningitis (CM) diagnoses increased. (Fig 2).

**Table 3:** Meningitis etiologies and outcomes per group.

	Group 1 n = 321	Group 1 Mortality n = 127	Group 2 n = 890	Group 2 Mortality n = 278	Group 3 n = 232	Group 3 Mortality n = 389	Total Cases n = 1443	Total Mortality n = 494
	n, (%)	n (%)	n (%)	n, % 278	n,(%)	n (%)	n (%)	n (%)
**Confirmed CM**	44/321 (13.7)	25 (56.8)	172 (19.6)	67 (38.5)	36 (15.5)	9, (25.0)	252 (17.5)	101 (40.1)
**Presumed CM**	84 (26.2)	31 (36.9)	85 (9.6)	33 (39.8)	4 (1.7)	2 (50.0)	173 1(2.0)	66 (38.2)
**Presumed & Confirmed CM**	128 (39.9)	56 (17.4)	259 (29.1)	100 (39.4)	40 (17.2)	11 (27.5)	427 (29.6)	167 (9.1)
**Confirmed BM**	0	NA	3 (0.3)	0	25 (10.8)	8 (32.0)	28 (1.9)	8 (28.6)
**Presumed BM**	0	NA	2 (0.2)	0	51 (22.0)	21 (41.1)	53 (3.7)	21 (39.6)
**Presumed & Confirmed BM**	0	NA	5 (0.6)	0	76 (32.8)	29 (38.2)	81 (5.6)	29 (35.8)
**Confirmed TBM**	0	0	1 (0.1)	1 (100)	9 (3.9)	6 (66.7)	10 (0.69	7 (70.0)
**Presumed TBM**	44 (13.7)	21 (47.7)	82 (9.2)	35 (42.7)	29 (12.5)	14 (48.3)	155 (10.7)	70 (45.2)
**Presumed & Confirmed TBM**	44 (13.7)	21 (47.7)	83 (9.3)	36 (43.4)	38 (16.4)	20 (52.6)	165 (11.4)	77 (46.7)
**Confirmed VM**	0	NA	0	NA	8 (3.5)	5 (62.5)	8 (0.55)	5 (62.5)
**Presumed VM**	2 (0.6)	1 (50)	17 (1.9)	4 (23.5)	40 (17.2)	13 (32.5)	59 (4.1)	18 (30.5)
**Presumed & Confirmed VM**	2 (0.6)	1 (50)	17 (1.9)	4 (23.5)	48 (20.7)	18 (37.5)	67 (4.6)	23, (34.3)
**MEM: CM + BM**	0	0	4 (0.4)	2 (50)	11 (4.7)	5 (45.5)	15 (1.0)	7 (46.7)
**MEM: CM + TBM**	0	NA	0	NA	2 (0.86)	2 (100)	2 (0.14)	2 (100)
**MEM: CM + VM**	0	NA	0	NA	2 (0.86)	0	2 (0.14)	0
**MEM: BM + TBM**	0	NA	0	NA	1 (0.43)	0	1 (0.07)	0
**MEM:BM + TBM + VM**	0	NA	0	NA	3 (1.3)	2 (66.7)	3 (0.21)	2 (66.7)
**MEM: BM + CM + VM**	0	NA	0	NA	1 (0.43)	1 (100)	1 (0.07)	1 (100)
**MEM Total**	**0**	**NA**	**4** (**0.4**)	**2**(**50**)	**20** (**8.6**)	**10** (**50.0**)	**24** (**2.1**)	**12** (**50.0**)
*Confirmed Total	**44** (**13.7**)	**25** (**56.8**)	**182 **(**20.4**)	**70 **(**38.5**)	**98 **(**42.2**	**38 (38.8**)	**324 (22.2)**	**131 **(**40.2**)
*Presumed Total	**92** (**28.7**)	**35** (**38.0**)	**186 **(**20.9**)	**72 **(**41.0**)	**49 **(**21.1**	**21 **(**42.9**)	**327 **(**17.9)**	**104 **(**40.3**)
**Presumed & Confirmed Total**	**136** (**42.4**)	**60** (**44.1**)	**368 **(**41.3**)	**142 (38.6**)	**147 **(**63.4**)	**59 **(**40.1**)	**651 **(**40.5)**	**235 **(**40.2**)
**Unknown**	**185** (**57.6**)	**67** (**36.2**)	**522 **(**58.7**)	**159 **(**30.5**)	**85 **(**36.6**)	**33 **(**38.8**)	**864 **(**59.9)**	**259 **(**30.0**)

CM: Cryptococcal meningitis; BM: Bacterial meningitis; VM: Viral meningitis; TBM: Tuberculosis meningitis; MEM: Mixed etiology meningitis.

* Some patients had confirmed and presumed meningitis, in such cases only confirmed total was designated.

Increased confirmed diagnoses for BM, TBM, and VM were identified in Group 3 after the introduction of additional diagnostic tests, including molecular tests in 2021 (S3 Table and Fig 2). Additionally, we observed more cases of multiple-etiology meningitis (MEM), defined as meningitis where ≥2 confirmed meningitis etiologies were identified, after the introduction of molecular testing. Twenty-cases of MEM were identified in Group 3, compared to four in Group 2, and most had CM as a contributing etiology (n = 16). In Groups 1 and 2, most meningitis diagnoses were presumed based on clinical impression as expanded diagnostic testing was not yet available. Increased availability of diagnostic tests over time improved diagnostic accuracy allowing etiology-targeted treatment plans instead of relying on clinical judgment. The increased availability and utilization of CSF cell counts for meningitis evaluation at LRRH allowed presumed cases of BM versus VM to be distinguished based on CSF pleocytosis and cellular differential counts. Thus, the number of confirmed plus presumed diagnoses increased over time. The number of unknown etiologies, defined as no confirmed or presumed diagnosis, decreased from 57.6% (Group 2) to 36.6% (Group 3) with the incorporation of additional diagnostics, although the specific etiology of the presumed cases was not defined. The specific etiologies of presumed VM cases remained unidentified.

### Etiologies of BM and VM

Pastorex, BioFire, culture, and gram stain identified *Streptococcus pneumoniae* as the most common BM etiology (Table 4). Culture allowed for the identification of other causes such as Klebsiella which is not a part of the molecular testing panels used. Culture also identified coagulase negative staphylococcus in two patients, which were potential contaminants. The identification of organisms not on the molecular panels supports the utility of culture which would allow identification of organisms that would be missed if molecular tests were used exclusively. We also identified viral etiologies in 14 patients through BioFire testing (Table 4). These etiologies included varicella zoster, cytomegalovirus, enterovirus, herpes simplex virus 1 (HSV-1), herpes simplex virus 2 (HSV-2), and human herpes virus 6 (HHV-6). However, viral etiologies which are not part of the BioFire panel would not be identified using our methods.

**Table 4:** Group 3 bacterial and viral etiologies and mortality.

Pathogen	BM n	Death n (%)	Pathogen	VM n	Death n (%)
Strep. pneumoniae	20	6 (30.0)	Varicella zoster	4	0
Neisseria meningitidis Y/W 135	3	1 (33.3)	Cytomegalovirus (CMV)	4	2 (50.0)
Escherichia. coli (E. coli)	4	2 (50.0)	Enterovirus	1	0
*Klebsiella	1	1 (100.0)	Herpes simplex 1 (HSV-1)	2	0
*Coagulase negative Staphylococcus	2	2 (100.0)	Human herpesvirus6 (HHV-6)	1	1 (100.0)
Strep. Pneumoniae + Neisseria meningitidis	1	1 (100.0)	HSV-1 + HHV-6	1	1 (100.0)
Gram positive cocci	10	3 (3.00)	Herpes simplex 2 (HSV-2)+HHV-6	1	1 (100.0)
**TOTAL**	**41**	**16 (39.0)**	**TOTAL**	**14**	**5 (35.7)**

*Organisms identified only through culture (likely a contaminant).

### Mortality

#### CM mortality improved over time, but overall meningitis mortality remained high.

The overall mortality for patients with confirmed CM was 56.8% in Group 1, 38.5% in Group 2 and 25.0% in Group 3 (Table 5). Combining confirmed CM patients with MEM (i.e., another confirmed etiology in addition to CM), the total CM mortality increased to 32.7% (n = 52) in Group 3. MEM mortality among CM patients was higher compared to those with CM only. In Group 1 the leading confirmed cause of death was CM, but few other diagnoses were confirmed. Confirmed diagnoses of BM, VM, and TBM were not made for Group 1 and for most patients the diagnosis was unknown.

**Table 5:** All HIV and non-HIV meningitis and mortality from Groups 1-3.

Confirmed Meningitis	HIV n = 1181	Non-HIV n = 136	HIV Mortality n = 414	Non-HIV Mortality n = 45
	**n (%)**	**n (%)**	**n (%)**	**n (%)**
**All Confirmed CM**	260 (22.0)	6/36 (4.4)	108/260 (41.5)	1/6 (16.7)
**All Confirmed BM**	30 (2.5)	14 (10.3)	12/30 (40.0)	4/14 (28.6)
**All Confirmed TBM**	12 (1.0)	3 (2.2)	8/12 (66.7)	2/3 (75.0)
**All Confirmed VM**	13 (1.1)	1 (0.7)	4/13 (30.8)	1/1 (100)
**TOTAL**	319 (7.1)	22 (16.2)	134/414 (32.4)	6/45 (13.3)
**All MEM**	23 (12.7)	1 (0.7)	11/23 (47.8)	1/1 (100)

From Group 3, we had the most accurate information about meningitis etiologies and mortality outcomes. Mortality was 32% for confirmed BM, 32.7% for confirmed CM (including MEM involving CM), and 66.7% for confirmed TBM. Patients with presumed BM had a mortality of 41.1% which was slightly higher than those with confirmed BM. Among 14 patients with confirmed and MEM VM, eight (57.1%) died and among 40 patients with presumed VM, 13 (32.5%) died. Mortality was highest among the 15 patients with confirmed TBM (66.7%) and the 29 patients with presumed TBM (48.3%).

Overall, 81.8% of meningitis patients across all groups were living with HIV (Table 1) and had higher mortality compared to those without underlying HIV infection (32.4% vs 13.3%) (Table 5). Among 44 patients with confirmed BM, 30 (68.2%) were HIV-infected with 40.0% mortality, compared to 28.6% mortality among those not living with HIV. Patients with confirmed VM, 13/14 had an underlying HIV infection with 30.8% mortality. In summary, an underlying HIV infection appeared to predispose BM, VM and TBM cases with higher mortality. Lastly, most patients with MEM (n = 23/24) were HIV-infected and had high mortality (47.8%). The 10 patients with MEM: CM + BM had 40.0% mortality, and all patients with MEM: CM + TBM died (Table 3).

## Discussion

Since implementing the CM-DTP in 2017 and transitioning to the MEN-DTP in 2021, we observed the expansion of molecular diagnostic testing led to increased confirmed diagnoses, allowing for improved targeted treatment of meningitis, especially CM. With these improvements, mortality steadily improved, most notably among those with CM. However, overall mortality remained high especially among PLWH with TBM and MEM. The introduction of new molecular diagnostics in 2021 (Group 3) enabled better identification of meningitis etiologies, including BM, VM, and TBM, which were rarely identified in Groups 1 and 2. Also, we discovered numerous patients who had MEM with more than one confirmed pathogen identified by molecular diagnostic testing. Overall, the implementation of this program provides insight into the prevalence of meningitis and the trend of dominant meningitis causes in rural, northern Uganda.

### Expanded diagnostics for meningitis improved identification of meningitis etiologies

The introduction of multiple diagnostic tests for meningitis enabled a comprehensive approach to improving the diagnostic yield. Diagnostic options such as CSF cell counts, cultures, and molecular tests including CrAg LFA, GeneXpert Ultra, Pastorex and BioFire were instrumental in confirming meningitis identification and enabled etiology- targeted treatments. Despite the well-established limitations of culture in the presence of antibiotic exposure, we found utility in performing CSF culture along with molecular testing [14–16]. Among those with confirmed meningitis, culture was third highest, behind BioFire and CrAg LFA, in meningitis identification. This finding was consistent from a study in Nigeria comparing the utility of bacterial culture and BioFire [17]. They concluded that BioFire was more rapid and sensitive in isolating bacterial agents in CSF, which was also supported from other studies [17–19]. The advantages of rapid PCR CSF testing using BioFire are evident; however, we recognize the added benefit of CSF culture as a complimentary diagnostic tool, as only selected bacterial pathogens included in molecular panels are identifiable using molecular diagnostic tests such as Pastorex and BioFire. Culture is not restricted to panel-specific pathogens, leading to broader diagnostic possibilities despite its limitations.

For confirmed CM identification, the CrAg LFA tests outperformed all other tests. Compared to other CM diagnostics, such as India ink, culture and BioFire, they had high concordance with CrAg LFA findings, but the CrAg LFA was more sensitive. India Ink was an outlier which identified five additional cases of CM that CrAg LFA did not capture, although false positive results cannot be excluded. Our findings support other studies that reported superiority of CrAg LFA for cryptococcus identification and CM diagnosis [20–22]. The low cost, ease of use, rapidity, and accuracy of this test underscore its importance as a diagnostic tool and support its transition to becoming the new gold standard for CM diagnosis [23].

BioFire is the only multiplex test available for VM diagnosis in Uganda. It is currently not a part of the routine clinical guidelines for meningitis testing [12], but it is more commonly incorporated into research studies conducted in the country. The cases of confirmed VM identified by BioFire in our study would have otherwise gone undiagnosed or depended on clinical diagnosis only. Among those with confirmed VM, most were due to common Herpes viruses such as Herpes Simplex Virus (HSV), Varicella Zoster Virus (VZV), and Cytomegalovirus (CMV). Many causes of viral meningoencephalitis, such as West Nile virus, Zika virus, Epstein-Barr virus, Coxsackie and Echovirus, are not present on the BioFire panel and would not be identifiable with BioFire testing. HIV itself can cause an aseptic meningitis, usually presenting at the time of seroconversion but does not usually cause the type acute meningitis syndrome that our patients experienced [24,25]. Our finding of large numbers of patients with presumed VM based on high cell counts with lymphocytic predominance suggests that other viral etiologies are likely causing disease in the region. The low diagnostic yield of confirmed VM in our study, suggests further studies with more sensitive diagnostics and expanded diagnostic capabilities would provide a clearer understanding of the true burden of VM among this rural population in Uganda.

The incorporation of TB GeneXpert Ultra in CSF testing provided crucial information about the true incidence of TBM at LRRH. All TBM diagnosis prior to the utilization of this test was made by clinical determination or Ziehl-Neelsen (ZN) stain. ZN staining has a low sensitivity ranging from 50-60% and is dependent on the staining processes and expertise of the laboratory personnel [26–28]. GeneXpert Ultra on CSF samples has a diagnostic sensitivity of 63–72% [29–31]. Our program found that GeneXpert outperformed ZN staining by identifying seven times more cases (Table 3). However, the relatively low sensitivity of GeneXpert Ultra warrants consideration of potential cases that were not identified and future opportunities for improved diagnostics for TBM identification.

### Meningitis mortality was highest among PLWH with TBM or MEM

Overall mortality of confirmed BM and CM cases were similar, although mortality due to BM was higher in PLWH compared to those without HIV. Also, as a percentage of total meningitis cases, BM occurred more frequently in PLWH compared to those without HIV. The highest mortality due to meningitis in this study was among PLWH diagnosed with TBM or MEM. TBM had the highest mortality of all confirmed meningitis cases, which was also seen in other studies [32]. Systematic reviews reported that patients with TBM and HIV co-infection had higher mortality [32,33]. Although CSF culture of Mycobacterium tuberculosis is considered the gold standard for TBM diagnosis, the clinical utility of this test is compromised due to the extended growth time needed for culture (≥7 days) and the need for urgent treatment [34]. Challenges still exist around TBM diagnosis due to the paucibacillary nature of these organisms [31], and a multipronged approach to the diagnosis of TBM may be needed based on a composite reference standard of positive ZN smear microscopy, GeneXpert or mycobacterial culture [29,30,35]. CM predominantly affects PLWH and among those with other immunocompromised conditions [3]. Prior to the initiation of the CM-DTP, many patients died from CM due to prolonged delays in diagnosis and inadequate treatment. Patient outcomes improved significantly after the initiation of the CM-DTP, as evidenced by a 41% reduction in mortality compared to baseline levels (56.8% vs. 33.9%). Thus, with appropriate and early diagnosis, treatment, and monitoring, improved outcomes in CM mortality is possible [8,36]. Overall, the improved CM mortality we observed in Group 3 of 33.9% is better than reported pooled CM mortality from a meta-analysis of 44% [37].

We identified numerous cases of MEM, predominantly among PLWH and underlying CM. When compared with PLWH with CM only, MEM including CM had higher mortality (45% vs. 27.0%). Overall, patients with MEM had the second highest incidence of mortality after TBM. There are few studies that examine how and why MEM develops, but they have posited these co-infections are due to underlying immunodeficiency [38,39]. Other studies have also reported MEM/co-infections, especially between CM and TBM [38–40], but a study in Uganda reported more occurrences between CM and VM (11.1%) [40]. Our program also identified cases of CM and BM MEM, which had 40% mortality.

VM was primarily identified among PLWH in our study, which was consistent with another study which found VM predominately among this population [41]. We found that 54.5% of confirmed VM cases died during the hospitalization. In patients with presumed VM, mortality was 32.5%, suggesting that VM due to viruses not detected by our BioFire panel are important contributors to mortality. With continued focus and ongoing support for meningitis diagnosis, treatment and prevention, progress in meningitis mortality can be achieved as demonstrated by the improvements seen among the program’s CM population.

### New diagnostics are needed to improve diagnostic yield

The use of multiple diagnostic tests in each patient aided in the identification of meningitis pathogens, suggesting that a multipronged approach is needed to increase the diagnostic yield. However, many cases of suspected meningitis which remained unidentified reinforces the need for better diagnostic tests to improve comprehensive diagnosis. Continued preventative measures for meningitis should be employed to decrease incidence and improve patient outcomes. In Uganda, available diagnostics for meningitis include CSF culture, gram stain, wet prep, CSF cell count, ZN stain, India ink, CrAg LFA, and GeneXpert PCR, but most tests are only available at referral or national hospitals or private laboratories. Results from these tests take two to seven days, and supplies for these tests are often limited, reducing accessibility. In Uganda, CSF cell counts are often unavailable at referral hospitals and are only available at private laboratories and the national hospital. In many places, if patients are unable to pay, clinical diagnosis and management is implemented without a definitive diagnosis. The use of molecular tests that are unaffected by antibiotic exposure [42,43], such as Pastorex or BioFire, are part of standard practice in resource-rich countries [44,45] but are currently unavailable in Uganda, outside of study settings.

We found that modern diagnostics (PCR and antigen detection) were superior to standard methods to identify BM and VM; however, no single test was sufficient to identify all meningitis causes, as certain tests proved more effective in identifying specific pathogens, such as CrAg LFA for CM and BioFire was the only test able to identify VM. The use of point-of-care antigen detection tests including CrAg LFA, and Pastorex is time efficient and cost-effective for meningitis diagnosis and treatment [46,47]. There were some cases of concordance between tests, but for accurate identification of meningitis pathogens, combination diagnostics can increase the diagnostic yield. Other studies reported similar gaps in meningitis diagostics [48–50] and incorporated a new field of molecular testing using next generation DNA/RNA (metagenomic) sequencing (NGS) to allow real-time analysis to identify pathogens in a single assay [51,52]. NGS is neither affected by prior antibiotic use, nor is it limited to specific pathogens. Instead, it distinguishes whether the genes for a specific pathogen (bacterial, fungal, viral) are isolated from the specimen. Studies have shown the utility for this new generation of meningitis testing and its promise for future diagnosis of infectious diseases [48–50,53]. Further understanding of its feasibility and utility in a limited-resource setting is needed, but with new advances around NGS, it may become the standard comprehensive test needed for future meningitis identification.

### MEM is commonly found among PLWH and contributed to high mortality

The discovery of frequent MEM among our study population prompted the use of a multi-diagnostic package where all enrolled patients with underlying HIV infection were tested for CM, BM, VM and TBM. A single positive test is insufficient because MEM requires a comprehensive meningitis diagnosis package for proper evaluation. MEM was the third most common confirmed meningitis etiology, behind CM and BM which highlights the hidden burden of this phenomenon and the importance of comprehensive diagnosis as a missed diagnosis can be fatal. Although MEM has been reported in other studies [38,54], our use of multiple diagnostic tests in each patient allowed us to identify MEM frequently in our patient population, From the confirmed meningitis cases found in Group 3, 8.6% had MEM and had worse mortality outcomes than those with a single meningitis etiology. Additionally, most MEM cases had underlying HIV infection. Few studies have assessed the factors associated with MEM, but some studies reported that interactions from general co-infections can occur on multiple levels including stress, severity, and immunological response modulators [55,56]. It is unknown which organism contributed to the symptoms and whether one infection unmasked the other. Further studies are needed to understand the dynamics and mechanisms of MEM through cytokine profiling, immunomodulation and regulatory responses.

### Limitations

There were limitations to our study related to the use of secondary data which relies on the documented data from the original source files. For Groups 1 and 2 few diagnostics were used to confirm meningitis, therefore most cases were based on clinical symptomology and provider impressions. This distinction was captured in our data which categorized clinical diagnosis as presumed meningitis. Due to limited data on specific organisms isolated we limited our analysis for BM, VM, TBM and confirmed CM diagnostics to Group 3 only. Among patients with MEM, we were unable to make associations regarding which organism contributed to death or survival due to our small sample size and lack of immune response investigations. Additionally, we cannot make assertions on MEM risks related to mortality such as type of meningitis due to our limited sample size.

## Conclusion

We have shown that a diagnostic package approach for meningitis is essential for improving comprehensive identification of meningitis etiologies. Although we did not find a single diagnostic test to recommend for comprehensive meningitis identification, molecular tests outperformed conventional testing methods and should be widely available for proper identification and accurate treatment. Further exploration in NGS should be conducted for feasibility and utility in resource-limited setting to become the single diagnostic needed for comprehensive meningitis diagnosis, the absence of routine vaccines for meningitis in northern Uganda has stymied the collective efforts of the World Health Organization, PATH, and other institutions to eliminate this disease. Community members continue to succumb to vaccine-preventable meningitis, especially among those with underlying HIV, for whom routine immunization should be included as part of the advanced HIV toolkit. Lastly, further investigation is needed to understand MEM and how underlying HIV infection, immune modulators and inflammatory markers contribute to patient treatment responses and outcomes. Leveraging proven prevention measures and modern diagnostics to gain further understanding of immune responses to meningitis will enhance our current diagnosis, treatment, and care to ultimately eliminate meningitis-related death and disability.

## Supporting information

S1 Table Available diagnostics and treatments in Groups 1–3. (DOCX)

S2 Table Pathogens identified by BioFire and Pastorex. (DOCX)

S3 Table Diagnostics utilized in Groups 1–3. (DOCX)
