# Supplementary material for: Meningitis diagnosis, treatment, and outcomes in rural, northern Uganda: 2015–2024
Source: PLOS Glob Public Health. 2026 Jan 12;6(1):e0005800. doi: 10.1371/journal.pgph.0005800 (PMC12795354; doi:10.1371/journal.pgph.0005800)
Supplement: S1 Table — (DOCX) [file pgph.0005800.s001.docx]

**Table 1. Available diagnostics and treatments in groups 1-3**

|  | | | |
| --- | --- | --- | --- |
|  | **Group 1** | **Group 2** | **Group 3** |
| Diagnostics | India ink (private lab) | India ink (private lab) | India ink (LRRH) |
|  | CrAg LFA (private lab) | CrAg LFA | CrAg LFA |
|  | CSF analysis/cell count (private lab) | CSF analysis/cell count (private lab) | CSF cell count |
|  | CSF culture (private lab) | CSF culture (private lab) | CSF analysis (LRRH) |
|  |  |  | CSF culture (LRRH) |
|  |  |  | BioFire |
|  |  |  | Pastorex |
|  |  |  | GeneXpert |
| Treatments | Deoxycholate Amphotericin B (private pay) | Deoxycholate Amphotericin B | Deoxycholate Amphotericin B |
|  | Fluconazole (private pay) | Fluconazole | Liposomal Amphotericin B |
|  | Magnesium (private pay) | Magnesium | Fluconazole |
|  | Potassium (private pay) | Potassium | Flucytosine |
|  | Ondansetron (private pay) | Ondansetron | Ceftriaxone |
|  | Routine CBC, CR, K (private pay) | Routine CBC, CR, K | Magnesium |
|  |  |  | Potassium |
|  |  |  | Ferrous sulfate |
|  |  |  | Ondansetron |
|  |  |  | Routine CBC, CR, K |
