# Supplementary material for: Meningitis diagnosis, treatment, and outcomes in rural, northern Uganda: 2015–2024
Source: PLOS Glob Public Health. 2026 Jan 12;6(1):e0005800. doi: 10.1371/journal.pgph.0005800 (PMC12795354; doi:10.1371/journal.pgph.0005800)
Supplement: S2 Table — (DOCX) [file pgph.0005800.s002.docx]

**Table 2. Pathogens Identified by BioFire and Pastorex**

|  | | |
| --- | --- | --- |
|  | **BioFire PCR** | **Pastorex LA** |
| Bacteria | *Escherichia coli* K1 | *Escherichia coli* K1 |
|  | Haemophilus influenzae b | *Haemophilus influenzae* b |
|  | *Streptococcus agalactiae* | Group B Streptococcus |
|  | *Neisseria meningitidis* | *Neisseria meningitidis* (A, B, C, Y/W135) |
|  | Streptococcus pneumoniae | *Streptococcus pneumoniae* |
|  | *Listeria monocytogenes* |  |
| Yeast | Cryptococcus (*C. neoformans/C. gattii*) |  |
| Viruses | Cytomegalovirus (CMV) |  |
|  | Enterovirus (EV) |  |
|  | Herpes simplex virus 1 (HSV-1) |  |
|  | Herpes simplex virus 2 (HSV-2) |  |
|  | Human herpesvirus 6 (HHV-6) |  |
|  | Human parechovirus (HPeV) |  |
|  | Varicella zoster virus (VZV) |  |
