# Supplementary material for: Meningitis diagnosis, treatment, and outcomes in rural, northern Uganda: 2015–2024
Source: PLOS Glob Public Health. 2026 Jan 12;6(1):e0005800. doi: 10.1371/journal.pgph.0005800 (PMC12795354; doi:10.1371/journal.pgph.0005800)
Supplement: S3 Table — (DOCX) [file pgph.0005800.s003.docx]

**Table 3. Diagnostics utilized in Groups 1-3**

|  | | | | | | |
| --- | --- | --- | --- | --- | --- | --- |
|  | **GROUP 1 Positive** | **Total n=321** | **GROUP 2 Positive** | **Total n=890** | **GROUP 3 Positive** | **Total n=232** |
| **Serum CrAg** | 37 (90.2) | **41 (12.8)** | 198 (31.5) | **628 (70.6)** | 53 (31.0) | **171,(73.7)** |
| **CSF CrAg** | 34 (29.8) | **114, (35.5)** | 162 (39.3) | **412 (46.3)** | 49 (31.2) | **157 (67.7)** |
| **India Ink** | 19/50 (38.0) | **50, (15.6)** | 72(57.1 ) | **126 (14.2)** | 20 (11.6) | **173 (74.6)** |
| **GeneXpert** | 0 | **NA** | 0 | **NA** | 14 (7.0) | **199 (85.8)** |
| **ZN stain** | 0 | **NA** | 1/1 (100) | **1 (0.01)** | 1 (1.1) | **95 (40.9)** |
| **Gram stain** | 20 (95.2 | **21 (6.5)** | 60 53.6) | **112 (12.6)** | 40 (18.8) | **213 (91.8)** |
| Yeast | 20 (95.2) | **21, (6.5)** | 56 (6.3) |  | 19 (8.2) |  |
| Bacteria | 0 | **NA** | 4 (0.4) |  | 24 (10.3 |  |
| **Cell Count** | 0 | **NA** | 362.3 (5.0-236.2) | **362.3 (5.0-236.2)** | 1233 (10-425) | **1233 (10-425)** |
| **Culture** | 0 | **NA** | 1/1 (100) | **1 (0.1)** | 30 (14.6) | **205 (88.5)** |
| **Pastorex** | 0 | **NA** | 0 | **NA** | 23/164 (12.0) | **164 (70.7)** |
| **BioFire** | 0 | **NA** | 0 | **NA** | 56/195 (28.7) | **195 (84.1)** |
| *CrAg: cryptococcal antigen*  *ZN: Ziehl-Neelsen* | | | | | | |
